# Clinical Outcomes among Hospitalized COVID-19 Patients Who Received Baricitinib or Tocilizumab in Addition to Standard of Care

**DOI:** 10.3390/diseases12050107

**Published:** 2024-05-20

**Authors:** Cucnhat P. Walker, Natalie P. Hurlock, Subrata Deb

**Affiliations:** 1 HCA North Cypress Medical Center, Cypress, TX 77429, USA; 2 HCA Healthcare Graduate Medical Education, Brentwood, TN 37027, USA; 3 Department of Pharmaceutical Sciences, College of Pharmacy, Larkin University, Miami, FL 33169, USA

**Keywords:** COVID-19, SARS-COV-2, coronavirus disease, tocilizumab, baricitinib, mortality, comparison

## Abstract

COVID-19 infection is caused by the novel severe acute respiratory syndrome coronavirus 2 (SAR-CoV-2). This novel virus has transformed into different resistant variants (e.g., omicron; delta; alpha; epsilon) since its first emergence in 2019. The National Institutes of Health and Infectious Diseases Society of America guidelines currently recommend adding either baricitinib or tocilizumab to the standard of care for severe COVID-19 treatment. An outcome comparison between baricitinib and tocilizumab is needed to determine which agent is more appropriate and safer in clinical practice when deciding treatment. We aimed to compare mortality and clinical outcomes between tocilizumab and baricitinib in the management of severe COVID-19 infection. A total of 5638 adult patients from 16 acute care hospitals in a large healthcare system in Texas were included in this multicentered retrospective cohort study. The median age of the patients was 56 years and 46.67% of them were female. Severe COVID-19 patients were treated with standard of care and either tocilizumab or baricitinib. The primary outcome of hospital admission mortality rates was found to be higher with tocilizumab (odd ratio (OR) of 1.56; *p* = 0.001; 95% CI 1.19 to 2.008) compared to that with baricitinib (OR 0.65; *p* = 0.001; 95% CI 0.50 to 0.84). For one of the secondary outcomes, patients who received tocilizumab were 3.75 times more likely to be admitted to the ICU than those receiving baricitinib (*p* = 0.001; 95% CI 2.89 to 4.85). Among the 1199 COVID-19 patients who were admitted to the ICU, the ICU length of stay was shorter among patients receiving baricitinib with a mean difference of 4.42 days and a median difference of 2.54 days, compared to those receiving tocilizumab (*p* < 0.0001; 95% CI −5.97 to −2.62) as another secondary outcome. Our large retrospective observational study showed that baricitinib reduced mortality; the likelihood of ICU admission; and the ICU length of stay compared to tocilizumab in patients with severe COVID-19 infection.

## 1. Introduction

Since the onset of the global coronavirus disease 2019 (COVID-19) pandemic in 2020, the disease surged again in 2021 and even higher in 2022. COVID-19 cases have been at a much lower number since 2023, with some peaks at certain times through the year. There seemed to be a rise in cases in the first half of January in 2024 based on the Centers for Disease Control and Prevention (CDC) [1]. There have been several therapies that have been tested and used and a large body of research evidence has been created in such a short period. Although the global pandemic seems to be behind us, sporadic severe COVID-19 cases still occur and the need to optimize effective treatment is ongoing, partly due to emerging viral resistance.

The Infectious Disease Society of America (IDSA) COVID-19 treatment guidelines version 11.0.0 currently recommend standard of care (SOC) for severe but non-critical disease to include corticosteroid (i.e., dexamethasone 6 mg intravenous (IV) or oral (PO) or the equivalent steroid for ten days) plus remdesivir for five to ten days [2]. SOC can be escalated to baricitinib for fourteen days or until discharged from the hospital, or a tocilizumab one-time dose is still part of the SOC in severe COVID-19 infection. Severe but non-critical disease is defined as an oxygen saturation (SpO_2_) of 94% in room air requiring high-flow nasal cannula (HFNC) oxygen or non-invasive ventilation (NIV). The National Institutes of Health (NIH) COVID-19 treatment guidelines currently also recommend the same standard of care [3].

Cytokine storm and its related inflammation are hallmarks of COVID-19 severity [4,5]. Interleukins have been a major driver of these inflammatory events during COVID-19 infection [6,7]. Interestingly, Janus kinase (JAK)-signal transducer and the activator of transcription (STAT) are strong stimulators of cytokines in COVID-19 [8,9]. Baricitinib is a selective JAK 1 and 2 inhibitor that blocks the intracellular signal pathway for the activation of certain inflammatory cytokines such as interleukin-2, interleukin-6 (IL-6), interleukin-10, interferon, and granulocyte–macrophage colony-stimulating factor [10,11]. This mechanism helps dampen the severe inflammation caused by the virus. It may also have direct antiviral activity [10]. Tocilizumab is a recombinant humanized monoclonal antibody that antagonizes IL-6 receptors, decreasing the production of cytokines and acute phase reactants. By inhibiting IL-6 receptors, tocilizumab similarly effectively reduces COVID-19 inflammation [10,12,13]. Both agents primarily treat COVID-19 patients by reducing severe inflammation.

The ACTT-2 trial demonstrated that baricitinib plus remdesivir proved to be superior to remdesivir alone in reducing recovery time (10 days versus 18 days) and accelerating clinical improvement among patients with COVID-19 needing HFNC oxygen or NIV at enrollment in the ACTT-2 trial [11]. However, the 28-day mortality benefit was not statistically significant in this trial. On the other hand, tocilizumab treatment showed a significant reduction in the 28-day mortality with a shorter median hospital LOS (19 days vs. >28 days) and was less likely to reach the composite endpoint of invasive mechanical ventilation or death in the RECOVERY trial [14].

The combination of both tocilizumab and baricitinib is not recommended in the current guidelines due to the need for more research evidence [2,3]. From the feedback provided by some of the prescribers at the hospitals included in this study and published reports, it can be inferred that it is a clinical dilemma for the prescribers to decide between baricitinib and tocilizumab, which indicates a need for certain criteria to initiate these agents [15]. These specific agents were chosen for comparison because they are the only options right now used to treat severe COVID-19. The clinical decision on which agent to prescribe was reported as whether the patient can take oral pills or not. Since the recommendation for their use was included in national guidelines, there has been a great need for head-to-head comparisons of these two agents, especially since these two agents are the only two options, if not the only options, to treat severe COVID-19 infection. In this large-scale, retrospective cohort study, we aim to compare mortality as the primary outcome and other secondary outcomes between baricitinib and tocilizumab in the treatment of severe COVID-19 infection.

## 2. Methods

### 2.1. Clinical Setup and Data Source

This study was approved by the Institutional Review Board of Hospital Corporation of America (HCA) Healthcare (Nashville, TN, USA) in October 2021. It is a large, retrospective cohort study of 5638 adult patients aged 18 years and older, with severe COVID-19 infection admitted to 16 HCA hospitals in the Houston area of Texas from 1 May 2021 to 30 September 2021 and who received either SOC or SOC plus at least one dose of baricitinib or tocilizumab. No patient was treated with both baricitinib and tocilizumab. The treatment protocol is standardized in all 186 HCA Healthcare hospitals across the United States. As per the hospital policy, all admitted patients were tested for COVID-19 via polymerase chain reaction (PCR). Treatment protocols were standardized and updated by the infectious disease clinical service corporate panel, based on available guidelines.

Tocilizumab or baricitinib can be considered for patients receiving dexamethasone and/or remdesivir with rapidly increasing oxygen needs (via conventional oxygen or HFNC or NIV or invasive oxygenation) and systemic inflammation. Tocilizumab was administered as a single weight-based dose intravenously infused over one hour and baricitinib was administered orally once daily for fourteen days or until hospital discharge, whichever came earlier. These agents were avoided in patients with known active tuberculosis or hepatic diseases (i.e., hepatitis B or C), pregnancy, or chronic immune-suppressing conditions. Tocilizumab was not used in patients with an active or high risk of bowel perforation including complicated diverticulitis. Table 1 describes the dosing criteria for tocilizumab based on the actual body weight of the patients and for baricitinib based on the patient’s renal function per corporate protocol. Pharmacists were authorized to monitor daily, adjust baricitinib dosage based on renal function, and hold therapy based on the laboratory criteria, as described in Table 1.

### 2.2. Data Collection and Outcomes

Data were extracted from the central corporate computer system based on pre-determined data points and not from individual patient chart reviews. The inclusion criteria included patients who tested positive for COVID-19 with severe clinical presentation and on SOC or SOC plus at least one dose of baricitinib or tocilizumab. All other patients in the database were excluded from this retrospective analysis. Due to the retrospective nature of this study, patients with all comorbidities and demography were included in this study. Baseline data points included demographics, pre-existing medical conditions, baseline vital signs within 24 h of admission, laboratory values, corticosteroids, and remdesivir therapy. Body mass index (kg/m^2^) was divided into four categories: (Cat) as <25 (Cat 1), ≥25 to <30 (Cat 2), ≥30 to <40 (Cat 3), and ≥40 (Cat 4).

The primary outcome was mortality. The secondary outcomes were hospital length of stay (LOS), time to intensive care unit (ICU) admission, and ICU LOS. The safety outcomes, including adverse drug reactions from baricitinib or tocilizumab and secondary bacterial co-infection post-bariticinib or post-tocilizumab therapy during hospital admission, were briefly reviewed for significant signals for the study period with submitted incident reports.

### 2.3. Statistical Analysis

Statistical analyses were performed using SAS 9.4 (Cary, NC, USA). Differences in patient baseline characteristics and pre-existing conditions between the treatment groups were evaluated using ANOVA and chi-square tests (Table 2). Multivariable regression models were used to evaluate the relationships between treatment type and patient outcomes (Table 3). Logistic regression models were used to evaluate the associations between treatment type and binary outcomes, including mortality and admission to the ICU. Linear regression models were used to evaluate the associations between treatment type and continuous outcomes, including time to ICU admission, ICU length of stay, and hospital length of stay. The following control variables were included in all multivariable regression analysis: age, sex, race, body mass index (BMI), hypertension, cardiovascular diseases, chronic lung diseases, diabetes mellitus, chronic kidney disease, and immunodeficiency. Using these methods, adjusted odds ratios (ORs) and adjusted mean differences (MDs) are presented with 95% confidence intervals. A *p* value of <0.05 was considered statistically significant.

## 3. Results

### 3.1. Baseline Characteristics

A total of 5628 adult patients diagnosed with severe COVID-19 infection were included in this study, of which 2631 patients (46.67%) were female. The ethnicity distribution was 3200 (56.76%) white patients, 804 (14.26%) black patients, and 1634 (28.98%) other. The median age of the cohort was 56 years with a range of 18 to 90 years. The median BMI was 31 kg/m^2^ with a range of 11.321 to 59.878 kg/m^2^, with BMI ≥25 to 30 kg/m^2^ being the highest at 39.75%. There were 4462 patients receiving SOC, while 738 patients received SOC plus baricitinib (BARI) and 438 patients received SOC plus tocilizumab (TOCI).

Table 2 describes the demographic characteristics and baseline variables of the cohort (SOC, BARI, and TOCI groups) with mean and median values obtained within 24 h of admission. Age, male gender, a history of cardiovascular disease, chronic lung disease, chronic kidney disease, diabetes mellitus, and immunodeficiency were found to be significantly associated with increased mortality for all three groups, while a BMI of ≥25 to <30 kg/m^2^ was found to have lower mortality compared to the three other BMI ranges among these patients. Overall, the baseline laboratory values within the first 24 h of admission, including oxygen saturation level, serum creatinine, alkaline phosphatase, ALT, AST, C-reactive protein (CRP), D-dimer, ferritin, high-sensitive troponin, white blood cell count (WBC), hemoglobin (Hgb), hematocrit (Hct), and platelets, were numerically higher in the BARI and TOCI groups than the SOC group. Statistical association was not performed on baseline laboratory values and mortality.

### 3.2. Study Outcomes

Table 3 describes the patients’ hospital course characteristics in the three groups (SOC, BARI, TOCI). There was a total of 1055 deaths (18.23%), of which 572 (10.15%) occurred in the SOC group, 276 (4.90%) in the BARI group, and 207 (3.67%) in the TOCI group. The average hospital LOS for all study groups was 8.99 days. A total of 1233 patients in all groups (21.87%) were admitted to the ICU during their hospitalization with the average time to ICU admission being 4.37 days and the average ICU LOS being 9.217 days. While the BARI group had the longest time to ICU admission, the TOCI group had the longest hospital and ICU LOS. The average time to the first dose of baricitinib or tocilizumab was 3.163 days. For the primary outcome, baricitinib therapy was found to have a statistically significant lower odds of mortality than tocilizumab therapy with OR 0.65 (95% CI 0.50 to 0.84, *p* 0.001). Compared to tocilizumab therapy, patients treated with baricitinib were found to have a significantly shorter hospital LOS of 3.75 days (95% CI −4.71 to −2.79, *p* < 0.0001), to be 0.27 times less likely to be admitted to the ICU (95% CI 0.20 to 0.35, *p* < 0.0001), to have a longer time to ICU admission of 2.66 days (95% CI 1.64 to 3.69, *p* < 0.0001), and to have a shorter ICU LOS of 4.29 days (CI −5.97 to −2.62, *p* < 0.0001).

## 4. Discussion

This study aimed to compare the clinical benefits between baricitinib and tocilizumab treatments among patients with severe COVID-19 infection, using SOC alone as the control. These patients tend to be older and have pre-existing diseases, specifically obesity, cardiovascular disease, chronic lung disease, chronic kidney disease, diabetes mellitus, and immunodeficiency. Levels of cardiac biomarkers (highly sensitive troponin and D-dimer), as well as inflammatory markers (CRP and ferritin), were numerically elevated, with higher levels especially among patients treated with baricitinib and tocilizumab, compared to the SOC. These findings were consistent with those of previous studies [7,16]. In addition, older male patients were found to have significantly higher mortality. Patients treated with baricitinib and tocilizumab appeared to have worse renal and hepatic functions compared to those treated with SOC.

After the results of the ACTT-2 trial and the RECOVER trial were published, there were a few trials examining the combination of baricitinib and tocilizumab published toward the end of 2021 and early 2022. Masia et al., in Spain, did not find a substantially reduced mortality by giving baricitinib to COVID-19 patients with clinical progression despite treatment with tocilizumab, corticosteroids, and/or remdesivir [17]. Among the 190 propensity score-matched patients in this study treated with baricinib plus SOC (tocilizumab plus corticosteroids) vs. SOC, some of whom also received remdesivir, there was no significant effect of baricitinib observed in the 28-day (adjusted HR 0.76, 95% CI of 0.31 to 1.86), 60-day (adjusted HR 1.17, 95% CI of 0.55 to 2.52), or 90-day mortality (adjusted HR 1.14, 95% CI of 0.45 to 1.72). Baricitinib addition was not associated with an increased risk of secondary infections (17.9 vs. 10.5%, *p* 0.212) or thromboembolic events (adjusted HR 1.89, 95% CI of 0.31 to 11.57, *p* = 0.490), especially after adjusting for age and sex. Another small, non-randomized, open-label Greek study by Gavriilidis et al. examined different COVID-19 regimens, including SOC, SOC plus tocilizumab, SOC plus anakinra, and SOC plus tocilizumab plus baricitinib [18]. The combined tocilizumab and baricitinib group was found to have a significantly lower 28-day mortality (*p* = 0.014), lower intubation rate (*p* = 0.013), and shorter duration of hospitalization (*p* = 0.019) compared to other treatment groups of patients with PaO_2_/FiO_2_ < 100 mmHg. It is worth noting that the nebulized dornase alfa treatment used, along with tocilizumab and baricitinib combination, make these findings difficult to interpret the clinical contribution from dornase alfa treatment. A systematic review and meta-analysis by Albuquerque et al. analyzed 27 randomized controlled trials and demonstrated a high probability that baricitinib was not inferior to tocilizumab [19]. However, this non-inferiority estimate was based principally on indirect evidence, which warranted cautious interpretation.

There have been some recent comparative, retrospective studies published in the past year focusing on tocilizumab vs. baricitinib in the treatment of severe COVID-19 stages. One study by Peterson et al., published in 2023, directly compared baricitinib vs. tocilizumab being added to SOC in the treatment of severe COVID-19 [20]. Among a total of 956 patients included, 665 receiving baricitinib were compared with 291 receiving tocilizumab, using propensity score matching. The study did not show a significant difference in the in-hospital mortality between the two agents. Other clinical outcomes, such as LOS less than 5 or discharge, change in LOS from day 1 to 14, hospital LOS, ICU LOS, and progression to MV or ECMO, were also similar. Adverse effects of secondary infections, thrombotic events, and acute liver injury were seen more frequently in the tocilizumab group; however, the frequency of these incidents was low. The study by Sunny et al. includes data over a longer period of one year and four months from a pool of 921 patients who received tocilizumab and 638 patients who received baricitinib, with a propensity-matched cohort of 597 patients in each group [21]. They found a quicker respiratory recovery (i.e., lower supplemental oxygen, NIV, or HFNC) at day 7 with the baricitinib group with no significant difference in 14-day and 28-day clinical status between the two groups. The 28-day mortality rate is only the secondary outcome reported in the overall group and it was shown to be significantly lower in the baricitinib group (tocilizumab 49% vs. baricitinib 39%, *p* = 0.001). Karolyi et al. (2021) showed that, among 159 patients with COVID-19 infection, hospital mortality, progression to MV, and hospital LOS were higher in the tocilizumab group but these results were not statistically significant at, respectively, 18% vs. 11% (*p* = 0.229), 19% vs. 11% (*p* = 0.173), and 13 vs. 12 days (*p* = 0.114) [22]. Two other similar but smaller studies also showed the same finding of no significant mortality rate. One of them was by Reid et al., using 176 patients infected with moderate to severe COVID-19, and reported a higher rate of adverse effects with tocilizumab treatment; the other one was by Kojima et al., using 98 patients with severe COVID-19 infection, and reported no significant difference in secondary infection rate [23,24]. A meta-analysis of 10 small studies was performed by Zhang et al. (2023); they found no significant difference in the 28-day mortality rate (OR 1.10, 95% CI 0.80 to 1.51, *p* = 0.57) and hospital LOS (OR −0.68, 95% CI −2.24 to 0.87, *p* = 0.39) between the two studied therapies, with the incidence of adverse effects (i.e., secondary infection rate, thrombotic and bleeding events, and acute liver injury) being higher with tocilizumab treatment. However, their calculated heterogeneity among the 10 studies was significant (*I*^2^ 57%, *p* = 0.01) and the sample sizes varied significantly, ranging from 52 to 582 patients, in addition to the fact that this study did not focus on one stage of COVID-19 severity [25].

Our study with a larger sample size, on the other hand, showed a more significant mortality benefit with baricitinib than with tocilizumab. However, we did not perform propensity score matching among the study groups. In addition to the mortality benefit, baricitinib was also found to have a significantly shorter hospital LOS, lower probability of progressing to ICU admission, and shorter ICU LOS. As Sunny et al. reported, the earlier respiratory recovery or the lower 28-day mortality rate by oral baricitinib can be explained partly by the steady serum drug level from the daily dosing of baricitinib in comparison to the declining serum drug level over time from the intravenous one-time dose of tocilizumab. This poses the question of whether it is beneficial for certain patients to receive repeated doses of tocilizumab and what risks to consider in doing so. Although we did not perform any statistical analysis on adverse outcomes from these two therapies, we did not observe any significant signal in reported adverse reactions from either agent among our study patients. Also, we did not thoroughly investigate secondary infections versus bacterial co-infections nor which patients in our study population received concurrent antibiotic treatments vs. antibiotic treatment initiated secondary to secondary infections from baricitinib or tocilizumab. Reporting adverse drug events (ADEs) from tocilizumab or baricitinib presents a great challenge due to multiple factors, including various and inconsistent monitoring and reporting from one hospital to the next, contributing factors of COVID-19 infection itself to different organ enzymes examined in these studies, different hospital practices and competency levels of staff in terms of secondary infections caused by contaminating intravenous access placement, close daily monitoring of drug therapy and appropriate dose adjustment, and frequency of laboratory ordering by prescribers. For patients’ baseline characteristics, most of our findings are consistent with previous publications such as older age, obesity, history of cardiovascular disease, chronic lung disease, chronic kidney disease, diabetes mellitus, and immunodeficiency being adversely associated with the severity of COVID-19. Interestingly, we also found that the male patients are 1.326 times more likely to have worse mortality than female patients and that being overweight (BMI of ≥25 to <30 kg/m^2^) was linked to lower mortality. Most patients with severe COVID-19 had a significantly decreased to no oral dietary intake. In addition, the national shortage of intravenous nutrition during the COVID surge causes temporary starvation periods among COVID-19 patients. These factors compounded the disease burden on the body. Carrying some extra body weight might provide these patients with some energy storage for utilization during times of sickness and starvation. Hence, being overweight was beneficial in severe COVID-19 infection. This statement still holds true even in the post-COVID-19 pandemic era as different hospitals have different nutritional practices, especially tube feeding and parenteral nutrition, which makes it beneficial to be slightly overweight. Elkhapery et al. (2024) showed no significant mortality difference among overweight patients with a BMI of 25 to <30 infected with COVID-19, while a higher BMI was linked to higher mortality [26]. Lastly, we did not perform any statistical analysis on baseline laboratory values; nonetheless, patients receiving SOC plus either of the study agents had numerically higher laboratory values than those treated only with SOC. This could be due to a faster clinical deterioration in these patients with higher laboratory values in the first 24 h of their admissions that prompted the prescribers to add baricitinib or tocilizumab to SOC. Lastly, during the COVID-19 surges, the stock availability of tocilizumab and baricitinib also contributed to the treatment decisions of the prescribers. Despite the ongoing mutation of the COVID-19 virus and viral resistance to treatment options, this study provides valuable findings for clinical practice in helping prescribers decide which treatment options should be initiated empirically upon patients’ hospitalization, as the major guidelines still include tocilizumab and baricitinib as part of the COVID-19 treatment pyramid.

The current study has several limitations, mainly due to the inherent biases in retrospective cohort studies, unmeasured confounding variables that may have influenced the results, and limitations in causal links in the subgroups. Firstly, due to the data being collected from a central corporate computer system with the predetermined data points being pulled from our patient sample, it was not possible to perform a comprehensive statistical analysis. Specifically, comparison analyses were not performed on laboratory values among the study groups. Some laboratory data, such as creatinine kinase-myoglobin binding (K-MB), N-terminal prohormone of brain natriuretic peptide (NT-proBNP), and myoglobin, were not included. In addition, we did not collect data on the median and mean days of baricitinib treatment. Nonetheless, these omissions should not have affected our study findings. Secondly, due to the data collection approach of pulling single data points from our computer system, we were unable to perform a propensity score-matched analysis with our results. Thirdly, although we did not observe any significant signal for reported ADEs from baricitinib and tocilizumab, such as secondary infections or infusion-related reactions with tocilizumab administration, we did not investigate deeply into concurrent antibiotic regimens. Our lack of ADE reporting does not equate to a lack of ADEs of these agents. Peterson et al.’s study found that adverse effects were lower with baricitinib than with tocilizumab in the propensity score-matched groups with an odds ratio (OR) of 0.6 (95% CI 0.4 to 0.8, *p* < 0.01). The occurrence rates of secondary infection, thrombotic events, and acute liver injury were also significantly lower with baricitinib. Baricitinib has a 12 h pharmacologic half-life vs. tocilizumab, for which it is up to 13 days. The median days of baricitinib treatment was 8. The authors suspected that the more prolonged drug effect might explain the higher incidence of ADEs with tocilizumab [20].

## 5. Conclusions

Our retrospective, observational study was conducted on 5628 patients hospitalized with severe COVID-19 who were treated with either SOC, SOC plus baricitinib, or SOC plus tocilizumab at multiple hospitals within a large health system in Texas. Baricitinib therapy was found to have a lower mortality, a shorter hospital and ICU LOS, a longer time to ICU admission, and to be less likely to progress to ICU admission. Furthermore, our study did not fully assess the adverse effects associated with these drugs of interest, which might have affected the findings.

## Figures and Tables

**Table 1:** Tocilizumab and baricitinib treatment protocols.

Drug Name	Tocilizumab	Baricitinib
Dosing criteria	ABW * > 90 kg: dose 800 mg ABW * 66–90 kg: dose 600 mg ABW * 41–65 kg: dose 400 mg ABW * 30–40 kg: 8 mg/kg ABW * < 30 kg: 12 mg/kg	GFR ^Ω^ ≥ 60: 4 mg GFR ^Ω^ 30–59: 2 mg GFR ^Ω^ 15–29: 1 mg GFR ^Ω^ <15: use not recommended
Criteria for holding therapy	ANC ^∞^ < 1000 cells/µL Platelet count < 50,000 cells/µL AST ^α^/ALT ^β^ > 10 times the upper limit of normal	ALC ^µ^ < 200 cells/µL ANC ^∞^ < 500 cells/µL AST ^α^/ALT ^β^ > 10 times the upper limit of normal

* ABW = actual body weight (kg). ^α^ AST = aspartate animotransferase (normal range 15–37 units/L). ^β^ ALT = alanine transaminase (normal range 6–50 units/L). ^µ^ ALC = absolute lymphocyte count (normal range 1.5–4.0 cells/µL). ^∞^ ANC = absolute neutrophil count (normal range 1.5–7.0 cells/µL). ^Ω^ GFR = glomerular filtration rate (mL/minute/1.73 m^2^).

**Table 2:** Baseline characteristic distribution (*n* = 5638).

Baseline Characteristics	SOC	SOC + Baricitinib	SOC + Tocilizumab	Chi-Square Test *p*-Value
	*n* (%)	*n* (%)	*n* (%)	
Sex:				<0.0001
Female (F)	2174 (38.56)	296 (5.25)	161 (2.86)	
Male (M)	2288 (40.58)	442 (7.84)	277 (4.91)	
Race:				<0.0001
White (W)	2473 (43.86)	514 (9.12)	213 (3.78)	
Black (B)	717 (12.72)	42 (0.74)	45 (0.80)	
Other (O)	1272 (22.56)	182 (3.23)	180 (3.19)	
BMI (kg/m^2^):				<0.0001
<25 (Cat 1)	831 (15.07)	62 (1.12)	32 (0.58)	
≥25 to <30 (Cat 2)	1245 (22.58)	168 (3.05)	97 (1.76)	
≥30 to <40 (Cat 3)	1650 (29.92)	196 (3.55)	196 (3.55)	
≥40 (Cat 4)	638 (11.57)	145 (2.63)	104 (1.89)	
Hypertension	1640 (29.09)	313 (5.55)	177 (3.14)	0.0067
Cardiovascular disease	1054 (18.69)	113 (1.65)	93 (2.00)	<0.0001
Chronic lung disease	823 (14.60)	187 (3.32)	97 (1.72)	<0.0001
Chronic kidney disease	563 (9.99)	78 (1.38)	43 (0.76)	0.0872
Diabetes mellitus	1767 (31.34)	329 (5.84)	206 (3.65)	0.0009
Immunodeficiency	146 (2.59)	17 (0.30)	18 (0.32)	0.2073
	**Mean (SD)**	**Mean (SD)**	**Mean (SD)**	**ANOVA *p*-value**
Age (years)	56.01 (17.42)	54.52 (14.94)	54.34 (14.57)	0.0190
SpO_2_ (%)	95.06 (3.98)	92.39 (3.49)	92.95 (4.03)	<0.0001
Serum creatinine (SCr) (mg/dL)	3.05 (10.24)	2.69 (8.91)	5.20 (18.48)	0.0002
Blood urea nitrogen (BUN) (4–23 mg/dL)	22.73 (19.69)	21.50 (13.11)	22.09 (15.66)	0.4439
Alkaline phosphatase (45–117 units/L)	88.87 (62.32)	84.02 (45.89)	88.99 (62.48)	0.1403
ALT (6–50 units/L)	62.81 (156.38)	73.60 (188.92)	62.55 (54.86)	0.2330
AST (15–37 units/L)	75.66 (228.73)	76.05 (72.41)	75.29 (61.84)	0.9980
CRP (0–0.33 mg/dL)	10.19 (7.74)	15.32 (9.62)	14.24 (8.61)	<0.0001
D-dimer (0–500 ng/mL)	2084.13 (4534.78)	1803.02 (3765.24)	2609.33 (5819.85)	0.0513
Ferritin (8–388 ng/mL)	864.92 (1122.62)	1348.06 (1049.20)	1112.32 (1076.99)	<0.0001
Troponin (0–78 pg/mL)	0.47 (3.85)	0.49 (3.78)	0.10 (0.38)	0.3407
WBC (4.5–11.0 × 10^3^/µL)	8.23 (4.99)	8.62 (3.85)	8.56 (3.98)	0.0662
Hgb (M 14.0–18.0 g/dL, F 12.0–16.0 g/dL)	12.95 (2.25)	13.65 (1.84)	13.68 (1.97)	<0.0001
Hct (M 40.0–55.0%, F 37.0–55.0%)	39.82 (6.27)	41.37 (4.98)	41.66 (5.49)	<0.0001
Platelet (150–400 × 10^3^/µL)	232.71 (98.21)	234.46 (85.22)	225.22 (78.85)	0.2499

**Table 3:** Hospital course characteristics and outcomes.

Outcomes	SOC	SOC + Baricitinib (BARI)	SOC + Tocilizumab (TOCI)	OR and *p*-Value
Deaths (%)	572 (10.15)	276 (4.90)	207 (3.67)	OR of 0.65 *p*-value = 0.001 (BARI vs. TOCI) and OR of 0.13 and *p*-value <0.0001 (SOC vs. TOCI)
ICU admission	728 (12.91)	231 (4.10)	274 (4.86)	<0.0001
Time to ICU admission (days)				<0.0001
Mean	3.22	7.57	4.76	
Median	1.00	7.00	3.00	
ICU LOS (days)				<0.0001
Mean	6.99	10.03	14.45	
Median	3.89	7.90	10.44	
Hospital LOS (days)				<0.0001
Mean	7.01	15.07	18.86	
Median	5.00	12.00	15.00	

## Data Availability

Data are contained within the article.
